# Dynamics of Short-Term Metabolic Profiling in Radish Sprouts (*Raphanus sativus* L.) in Response to Nitrogen Deficiency

**DOI:** 10.3390/plants8100361

**Published:** 2019-09-23

**Authors:** Seung-A Baek, Kyung-Hoan Im, Sang Un Park, Sung-Dug Oh, Jaehyuk Choi, Jae Kwang Kim

**Affiliations:** 1 Division of Life Sciences and Bio-Resource and Environmental Center, College of Life Sciences and Bioengineering, Incheon National University, Incheon 22012, Korea; bsa1103@inu.ac.kr (S.-A.B.); khim61@inu.ac.kr (K.-H.I.); 2 Department of Crop Science, Chungnam National University, 99 Daehak-ro, Yuseong-gu, Daejeon 34134, Korea; supark@cnu.ac.kr; 3 National Institute of Agricultural Sciences, Rural Development Administration, Wanju-gun, Jeollabuk-do 55365, Korea; ohbaboh@korea.kr

**Keywords:** nitrogen, nitrogen metabolism, nitrogen deficiency, radish sprouts, metabolic profiling

## Abstract

Nitrogen (N) is a macronutrient important for the survival of plants. To investigate the effects of N deficiency, a time-course metabolic profiling of radish sprouts was performed. A total of 81 metabolites—including organic acids, inorganic acid, amino acids, sugars, sugar alcohols, amines, amide, sugar phosphates, policosanols, tocopherols, phytosterols, carotenoids, chlorophylls, and glucosinolates—were characterized. Principal component analysis and heat map showed distinction between samples grown under different N conditions, as well as with time. Using PathVisio, metabolic shift in biosynthetic pathways was visualized using the metabolite data obtained for 7 days. The amino acids associated with glucosinolates accumulated as an immediate response against –N condition. The synthesis of pigments and glucosinolates was decreased, but monosaccharides and γ-tocopherol were increased as antioxidants in radish sprouts grown in –N condition. These results indicate that in radish sprouts, response to N deficiency occurred quickly and dynamically. Thus, this metabolic phenotype reveals that radish responds quickly to N deficiency by increasing the content of soluble sugars and γ-tocopherol, which acts as a defense mechanism after the germination of radish seeds.

## 1. Introduction

Radish (*Raphanus sativus* L.), a member of the Brassicaceae family, is consumed as an edible root vegetable throughout the world. The entire plant of radish, including leaves and sprouts, is edible. It contains anthocyanins, carotenoids, flavonoids, glucosinolates, policosanols, tocopherols, and phytosterol [1,2,3]. Radish sprouts also have anthocyanins, carotenoids, flavonoids, and glucosinolates, and their antioxidant properties have previously been demonstrated [4,5,6,7,8]. To enhance the quality of radish and to increase the contents of these phytochemicals, few fertilization methods have been investigated. The iodine fertilization was reported to increase the contents of ammonium ion and amino acids in radish roots [9]. Zhou et al. (2013) [6] investigated the effects of sulfur fertilization on the content of health promoting phytochemicals—such as glucosinolates, carotenoids, chlorophylls, and total phenolics—in radish sprouts.

Plants need macro- (nitrogen, phosphorus, potassium, calcium, magnesium, and sulfur) and micronutrients (boron, copper, iron, manganese, molybdenum, and zinc) for their survival; these nutrients are required for respiration, photosynthesis, signaling, and growth. For example, potassium is required for ensuring the osmotic balance, calcium maintains the structure of cell wall, and magnesium is a constituent of chlorophyll molecules. Among macronutrients, nitrogen (N) is important for the biosynthesis of chlorophylls, proteins, nucleic acids, and hormones [10]. Because of the important role of N, several studies have been conducted to investigate the effects of N supplementation and deficiency on plant physiology. In watercress grown under conditions of N supplementation, the chlorophyll and carotenoid contents were reported to increase [11]. Under N deficient (–N) condition, the concentration of N was reduced in the shoots and roots of rice. Under –N condition, the biomass and chlorophyll content of rice were reduced, and the length of roots was increased compared to that under N sufficient condition [12]. In *Arabidopsis* plants grown under –N condition, the total protein, and chlorophyll content, and fresh weight were decreased, and the content of anthocyanins, primary root length, and lateral root number were increased [13,14]. In cabbage, the growth was inhibited under –N condition, and the levels of most amino acids and organic acids were decreased compared to that under normal conditions [15].

Metabolic profiling can provide links and relationships that are revealed primarily through regulation at the metabolic level. Thus, it can potentially be used for determining the phenotype depending on metabolite changes [16,17,18]. Metabolic profiling has been performed to assess the response to N deficiency in many plants, such as tobacco, tomato, watercress, maize, rice, *Arabidopsis*, cabbage, and radish [11,13,19,20,21,22]. Especially, response to N and its regulation has been studied using genomics, transcriptomics (including microRNA), and proteomics tools, in *Arabidopsis* [13,14,23,24,25]. In previous studies, the effects of long-term N deficiency in seedlings or N limitation in mature plants have been characterized. In the present study, we conducted metabolic profiling of radish sprouts to investigate the short-term metabolic changes after germination of radish seeds under complete nitrogen starvation. Using gas chromatography-time-of-flight mass spectrometry (GC-TOFMS), gas chromatography-quadrupole mass spectrometry (GC-qMS), and high-performance liquid chromatography (HPLC), primary and secondary metabolites were detected during the –N treatment period. The knowledge about the changes in metabolites and differences in their contents between radish sprouts grown under N sufficient and –N conditions would provide fundamental information for understanding the N response system.

## 2. Results and Discussion

### 2.1. Phenotypic Distinction under Nitrogen Deficiency

**Figure 1:**
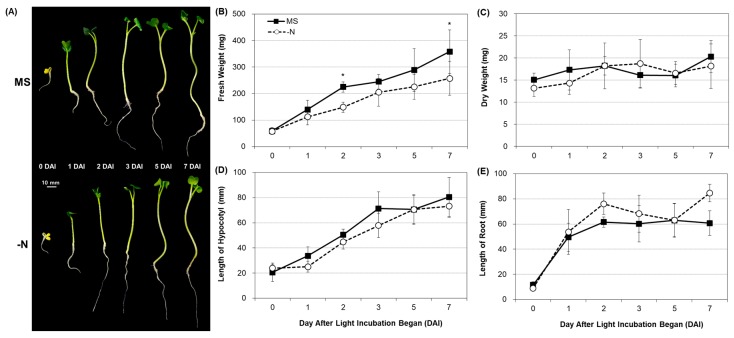
Phenotypic changes in radish sprouts during nitrogen deficient condition. (**A**) Growth of radish sprouts under nitrogen sufficient (MS) and deficient (–N) conditions. (**B**) Fresh weight and (**C**) dry weight of radish sprouts grown under MS and –N conditions. Changes in the length of hypocotyl (**D**) and root (**E**) of radish sprout grown under the two nitrogen conditions. Data for different points are the means ± SD (n = 6). * *p* ≤ 0.05. DAI, day after light incubation began.

Under the N limited condition, the growth and biomass of shoots were reduced, and the growth of roots was enhanced in *Arabidopsis*, cabbage, and rice [12,13,14,15,24]. In this study, under –N condition, the fresh weight of radish sprouts was reduced as in previous studies. However, there was no difference in the dry weight during –N treatment. The length of hypocotyl was slightly reduced under –N condition but was not statistically different from that under N sufficient condition, except at 1 day after light incubation began (DAI). As in other plants, the root length of radish sprouts was slightly increased under –N condition (Figure 1 and Table S1).

### 2.2. Metabolite Profiling of Radish Sprouts

We performed metabolite profiling of radish sprouts grown under N sufficient and –N conditions. A total of 81 primary and secondary metabolites were identified in radish sprouts using GC-TOFMS, GC-qMS, and HPLC. We identified 10 organic acids, 1 inorganic acid, 20 amino acids, 9 sugars and sugar alcohol, 2 amines, 1 amide, and 2 sugar phosphates in the samples using GC-TOFMS (Tables S2, S3, and Figure S1). Nine kinds of policosanols, three kinds of tocopherols, and five types of phytosterols were identified using GC-qMS (Tables S4 and S5). Policosanols are components of plant waxes and oils. Tocopherols have antioxidant activity and phytosterols are essential components of cell membranes in plant [26]. Docosanol (C22), α-tocopherol, campesterol, and β-sitosterol were predominant compounds in radish sprouts, which was in agreement with the results of previous research [21]. Eight kinds of carotenoids were detected using HPLC (Tables S6, S7, and Figure S2). The predominant carotenoids were lutein and β-carotene in radish sprouts incubated under the two growth conditions. Zeaxanthin was only detected at 0 and 1 DAI, but not thereafter. Carotenoids are photoprotective accessory pigments and antioxidant agents in plants. Glucosinolates are protective compounds that are released when plants are attacked by herbivores and get wounded. In all the samples, five kinds of aliphatic glucosinolates and four kinds of indolic glucosinolates were identified by HPLC (Tables S8, S9, and Figure S3).

### 2.3. PCA and Heat Map

**Figure 2:**
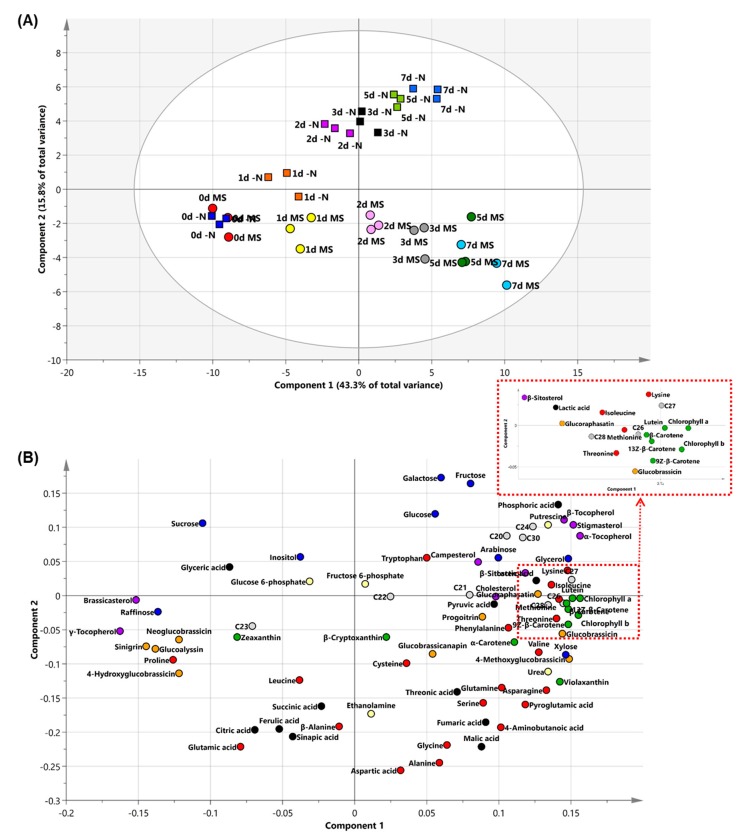
(**A**) Principal component analysis (PCA) score plots and (**B**) loading plots obtained from metabolite data of radish sprouts. PC1 and PC2 accounted for >59.1% of the total variance. Amino acids, pigments, and glucosinolates are represented as red, green, and orange, respectively; sugars and sugar alcohols are represented as blue; organic acids and inorganic acids are represented as black; policosanols are represented as gray; tocopherols and phytosterols are represented as purple; and amines, amides, and sugar phosphates are represented as yellow. d, day after light incubation began; MS, radish sprouts grown under nitrogen sufficient conditions; –N, radish sprouts grown under nitrogen-deficient conditions; C20, Eicosanol; C21, Heneicosanol; C22, Docosanol; C23, Tricosanol; C24, Tetracosanol; C26, Hexacosanol; C27, Heptacosanol; C28, Octacosanol; C30, Triacontanol.

The PCA (principal component analysis) was performed to identify class differences among multivariate data obtained from radish sprouts grown under different nutrient conditions (N sufficiency and deficiency) (Figure 2A). The result of PCA analysis showed that the pattern of metabolite changed with the growth of sprouts and revealed a clear difference in the metabolite profile between the two conditions. The first component (principal component 1; PC1) showed the change in primary and secondary metabolites when the sprouts were growing. The contents of amino acids—such as lysine, methionine, threonine, isoleucine, valine, and phenylalanine—were increased during the development. On the other hand, the contents of proline and leucine were decreased (Figure 2B, Tables S2 and S3). The contents of α- and β-tocopherol, phytosterols, except for brassicasterol, and policosanols, except for tricosanol (C23), increased with the growth. In canola, the change from γ-tocopherol to α-tocopherol during germination was observed, but there was no significant change in the composition of phytosterols [26]. In this study, the level of γ-tocopherol in radish sprouts was decreased and that of α-tocopherol was increased with the growth. As in canola, the change from γ-tocopherol to α-tocopherol seemed to occur during the growth of radish sprouts. The total content of policosanols and tocopherols was increased with the growth of radish sprouts under both the N treatment conditions. The total content of phytosterols was increased although brassicasterol was decreased during the growth of sprouts (Tables S4 and S5). Chlorophylls and carotenoids (carotenes, lutein, and violaxanthin) were accumulated as the sprouts grew. Chlorophylls and carotenoids are essential and accessory pigments, respectively, for photosynthesis. It appears that during germination, radish uses nutrients to make the first leaves, and with further growth, plants accumulate the pigments for photosynthesis (Tables S6 and S7). The content of polysaccharides and disaccharides (raffinose and sucrose) was decreased, and that of monosaccharides (glucose, galactose, and fructose) increased with the growth. For use as energy sources, raffinose and sucrose were hydrolyzed to monosaccharides. The content of glucosinolates—except for sinigrin, glucoalyssin, 4-hydroxyglucobrassicin, and neoglucobrassicin—was also increased. In Brassicaceae, these four glucosinolates are biosynthesized through different pathways using different intermediates and genes [27]. It has been indicated that different pathways, involving several genes and enzymes, are used during the development of sprouts (Tables S8 and S9).

The PC2 separated samples grown under –N condition from those grown under N sufficient condition. At 0 DAI, the samples grown under the two N conditions could not be separated; however, with growth, the separation was obvious, indicating that the differences in metabolites were much larger than they were during the early stage of growth. In the loading plot, the significant metabolites of component 2 were amino acids (glutamic acid, alanine, aspartic acid, glycine, β-alanine, and 4-aminobutanoic acid), organic acids (malic acid, sinapinic acid, citric acid, ferulic acid), and sugars, such as glucose, fructose, galactose, and sucrose. It was indicated that radish sprouts grown under –N condition lacked amino acids, and organic acids, and accumulated more sugars compared to that under N sufficient condition (Figure 2B).

**Figure 3:**
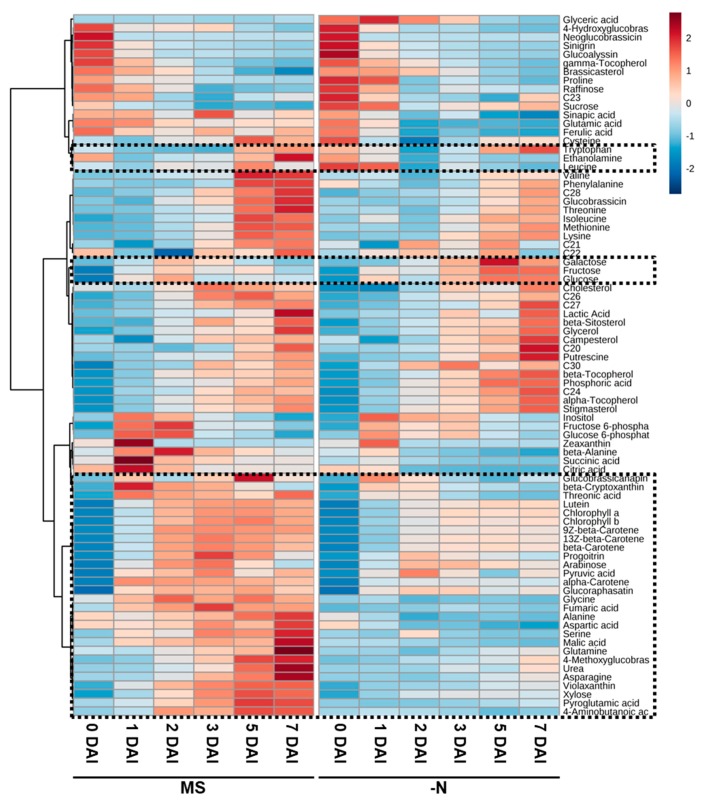
Heat map of differences in the contents of metabolites between radish sprouts grown under nitrogen sufficient (MS) and nitrogen deficient (–N) conditions. The average of standardized data is indicated in shades of red and blue for increase and decrease, respectively, in the metabolite content. DAI, day after light incubation began; C20, Eicosanol; C21, Heneicosanol; C22, Docosanol; C23, Tricosanol; C24, Tetracosanol; C26, Hexacosanol; C27, Heptacosanol; C28, Octacosanol; C30, Triacontanol.

To compare the changes in the individual metabolites during sprout development, heat map of metabolomics data, generated based on the average of standardized data, was used as a visualization tool (Figure 3). Consistent with the results of PCA, the pattern of changes in the metabolites, such as tocopherols and phytosterols, was also observed in the heat map. Moreover, metabolites, which showed different patterns of change in samples grown under N sufficient and –N conditions, were identified. For example, at 0 and 1 DAI, the levels of tryptophan and leucine in samples growth under N sufficient condition were lower than in samples grown under –N condition. Sucrose, galactose, fructose, and glucose were accumulated under –N condition. Pigments and glucosinolates were increased with the growth, but their levels were higher in samples grown under N sufficient condition than under –N condition (Figure 3, boxed within dotted lines).

### 2.4. Metabolic Shifts during the Development of Radish Sprouts under Nitrogen Deficient Condition

The result of PCA showed the overall pattern of metabolite and contributor to explain the effect of N during the growth of sprouts. The change and difference in the level of each metabolite under the different N treatment conditions were detected by heat map. PathVisio was used to confirm the comprehensive shift in metabolic pathways during the growth of sprouts under N sufficient and –N conditions. PathVisio is a free open-source biological pathway analysis software and has been used for comprehensive interpretation of large-scale experimental data [28].

To confirm the metabolic changes during the growth of radish sprouts, the log 2-fold change (log_2_ FC) values for metabolite levels at each DAI relative to the levels at 0 DAI were used. The downloaded WikiPathways [AtMetExpress overview (*Arabidopsis thaliana*)] was edited and the log_2_ FC (1 DAI/0 DAI) value, and those for other days (2 DAI/0 DAI, 3 DAI /0 DAI, 5 DAI /0 DAI, and 7 DAI /0 DAI), under the two growth conditions were applied to the pathway using PathVisio. The variations in the levels of metabolites were expressed as red and green, according to the scale bar (Figure S4). As for the loading plot of PCA, common pattern of changes in the contents of sugars, policosanols, tocopherols, phytosterols, pigments, and glucosinolates, were detected under both the nutrient conditions. However, PathVisio showed the differences between the samples grown under the two conditions in more detail. The levels of serine, glycine, cysteine, alanine, leucine, valine, β-alanine, aspartic acid, glyceric acid, malic acid, and succinic acid in the –N samples were reduced with the growth of radish sprouts, whereas the levels in the control samples were increased. These results only showed the pattern of change in metabolite levels in each sample with the growth of sprouts but did not provide a comparative assessment of the levels of metabolites in samples grown under different N treatment conditions.

**Figure 4:**
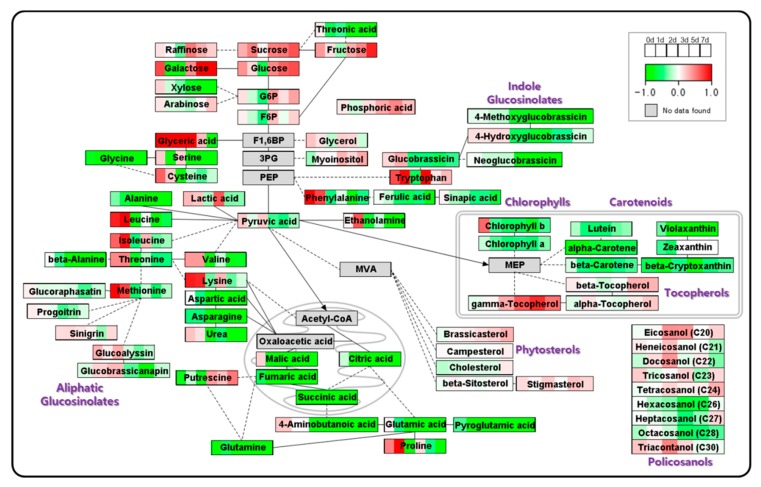
Metabolite change data mapped onto the schematic pathway of radish sprouts growth. The log 2-fold change (log_2_ FC) values of metabolite levels in radish sprouts grown under nitrogen deficient condition relative to those under nitrogen sufficient condition on each day. The log_2_ FC values are represented as a gradient and can be visualized per box on the top right (increased abundance is shown in red and decrease in abundance is shown in green). The gray boxes represent the metabolites that could not be detected. The solid-lines represent a direct link and dotted-lines represent an indirect link between the metabolites. The solid-line arrows show the transfer of metabolites to cell organelles. G6P, Glucose 6-phosphate; F6P, Fructose 6-phosphate; F1,6BP, Fructose 1,6-bisphosphate; 3PG, 3-Phosphoglyceric acid; PEP, 2-Phosphoenolpyruvate; MEP, Mevalonate; MVA, Mevalonic acid; DAI, day after light incubation began.

The differences in the levels of metabolites and the pattern of change are shown in Figure 4. The log_2_ FC values of metabolite levels in samples grown under –N condition relative to those in N sufficient conditions on each day were applied to the same pathway using PathVisio. Red color indicates high levels of metabolites under –N condition with respect to those under N sufficient condition, and green indicates low level according to the scale bar. The contents of the intermediates of TCA cycle, such as citric acid, succinic acid, fumaric acid, and malic acid, and of amino acids related to organic acids—such as aspartic acid, asparagine, glutamic acid, glutamine, and pyroglutamic acid—were less in sprouts grown under –N condition than in those grown under N sufficient condition at the initial time and thereafter. The low levels of fumaric and malic acids are consistent with those reported in a previous study [29]. These metabolites are downstream of pyruvic acid, which is produced by glycolysis. In transcriptomic study using *Arabidopsis*, 6-phosphofructokinase (at4g04040), and fructose-bisphosphate aldolase (at4g26530) genes were downregulated under severe N stress condition. Moreover, the gene for pyruvate kinase (at3g49060), the enzyme that catalyzes the final step of glycolysis, was downregulated under severe N condition [13]. In proteomic research using *Arabidopsis*, phosphofructokinase (at4g04040), fructose-bisphosphate aldolase (at2g21330, at4g38970), and phosphoglycerate kinase (at3g12780) are downregulated under N deprivation condition [14]. Phosphofructokinase converts fructose-6-phosphate to fructose-1,6-phosphate; fructose-bisphosphate aldolase converts fructose-1,6-phosphate to glyceraldehyde-3-phosphate; and pyruvate kinase converts phosphoenolpyruvate to pyruvate in the glycolysis pathway. Because of reduced glycolysis, the intermediates of TCA cycle were reduced under –N condition. In addition, in this study, carotenoids were decreased in response to N deficiency at the initial time. Organic acids related to TCA cycle and carotenoid metabolism are produced during photosynthesis, and they serve as C skeletons for amino acid biosynthesis and light-harvesting, respectively [30]. Thus, these results reveal that N assimilation and C metabolism are highly interconnected and the metabolic responses to N deficiency occur dynamically.

The levels of methionine, tryptophan, and phenylalanine, which are precursors of glucosinolates, were high at early time points and were lower under –N condition. Moreover, the contents of other amino acids (isoleucine, threonine, valine, and lysine), associated with glucosinolate biosynthesis, were statistically different (*p* ≤ 0.05) between the two nutrient conditions at 0 DAI (Tables S2 and S3). However, there were no significant differences in the levels of glucosinolates, except for glucoalyssin, at 1 DAI. The statistical difference in the level of glucosinolates, especially in indolic glucosinolates, occurred after 2 DAI. The total level of glucosinolates was slightly lower in radish sprouts grown under –N condition than in those grown under N sufficient condition (Tables S8 and S9). In 7-day old *Arabidopsis* seedling, the regulation of glucosinolate synthesis by microRNAs (miR826 and miR5090) via downregulation of alkenyl hydroxalkyl producing 2 (AOP2) transcript (at4g03060) under N starvation was investigated [23,31]. The changes in the levels of amino acids in a short period, relative to that of glucosinolates, followed by a decrease in the level of glucosinolates, showed the possibility of regulation of glucosinolate synthesis by microRNAs or other mechanisms in radish sprouts as a response to –N stimulation. The levels of glucosinolates were decreased in 3-week old leaves of *Arabidopsis* grown under –N condition, but were increased in the roots [25]. The N supplement induced the decrease of indole glucosinolates but did not influence the levels of aliphatic and aromatic glucosinolates in watercress [11]. In broccoli, the total level of glucosinolates was high under insufficient N supply [32]. This suggests that the response mechanism of glucosinolates to the N concentration is complex and differs depending on the developmental stage, organ, and species.

Wang et al. (2012) [14] reported that upregulation of S-adenosylmethionine synthetase 1 (SAM1) supports ethylene and auxin biosynthesis signaling and enhances root growth under N limited condition. Under –N condition, roots were more developed than they were under normal condition in this study. Accumulation of methionine and phenylalanine in sprouts grown under –N condition could be explained based on their roles as precursors of ethylene and auxin, respectively. The enolase (at2g36530), which converts 2-phosphoglycerate to phosphoenolpyruvate, was upregulated under N deprivation condition [14]. Because phosphoenolpyruvate is a precursor of tryptophan and phenylalanine, we guess that enolase was upregulated for production of the hormone.

The assimilation of N relative to glutamine synthetase/glutamate synthase (GS/GOGAT) cycle has previously been studied [33,34]. Sánchez et al. (2002) [35] reported the amino acid metabolism in French bean (decrease of proline at low N supplementation). The low level of glutamine and glutamic acid, and change in the level of proline at 1 DAI under –N condition might have occurred because of the relationship between N assimilation and GS/GOGAT cycle.

Glucose, fructose, and galactose were accumulated to higher levels in sprouts grown under –N condition than they were under N sufficient condition. α- and β-tocopherols were probably converted from γ-tocopherol in both the samples, but the level of γ-tocopherol was high under –N condition. The soluble sugars were increased in tomato and cabbage under –N condition [15,20]. Endogenous sugars are increased under N limited condition as a result of abiotic stress, and sugars induce the accumulation of anthocyanins [7]. Krapp et al. (2011) [36] reported that *Arabidopsis* roots and shoots show distinct temporal adaptation patterns toward N starvation. γ-tocopherol is one of the most potent antioxidant agents [37]. The high levels of sugars and γ-tocopherol act as defense mechanisms against N deficiency stress. The high level of putrescine in sprouts under –N condition was also suggested to be a response to stress [38]. Ascorbate is a precursor of threonic acid and is an antioxidant [39]. The high level of ascorbic acid was induced by decreased N applications in cabbage, cauliflower, and lettuce [40]. The low level of threonic acid under –N condition could be a result of the preferential use of ascorbate.

There was no distinguishable difference in the level of phytosterol in the two samples. β-sitosterol and stigmasterol are important for maintaining the structure and function of the cell membrane [41]. In the development of radish sprouts, it is guessed that the requirement of stigmasterol is more than that of β-sitosterol and brassicasterol. Campesterol is the precursor of brassinosteroid, which regulates the development and morphogenesis of plants [41]. Autophagy plays a role in resistance to abiotic stress, including N starvation [42,43], and a brassinosteroid-related autophagy mechanism was detected in *Arabidopsis*. Plants with the *atapg9-1* mutant gene, involved in autophagy, showed early chlorosis [44]. The 26S proteasome was degraded by autophagy in N starvation [45]. Brassinosteroid-induced autophagy occurred to reuse protein aggregates for increasing resistance to N starvation in tomato [46]. Because of the importance of brassinosteroids, the content of campesterol was maintained at high levels (Tables S4 and S5).

With regard to the pigments, there was only a difference in the content, but no change in the pattern was observed between the samples. Under –N condition, the levels of carotenoids and chlorophylls were low in samples grown under –N condition at 1 DAI and thereafter. The decrease in the content of pigments under –N condition has been well studied in many plants including kale, cabbage, rape, rice, sorghum, sweet potato, and tomato because of their relationship with photosynthesis and senescence [40,47,48,49,50,51].

## 3. Materials and Methods

### 3.1. Plant Materials and Culture Conditions

Radish seeds were purchased from Nongwoo Bio Co., Ltd. (Yeoju, Gyeonggi, Korea). The seeds were sterilized with 1% sodium hypochlorite containing 0.01% Tween 20 for 7 min, and 70% ethanol for 5 min. They were then rinsed five times with deionized water. The seeds were soaked by dipping in deionized water at 37 °C for 4 h and sown on 100 mL of modified Murashige and Skoog’s medium (with or without N) in Phytohealth (120 × 80 mm, SPL Life Sciences, Gyeonggi, Korea). The composition (per L) of nutrient sufficient medium was as follows: 0.825 g NH_4_NO_3_ (10.30 mM), 0.950 g KNO_3_ (9.40 mM), 0.085 g KH_2_PO_4_ (0.63 mM), 0.185 g MgSO_4_·7H_2_O (10.75 mM), 0.220 g CaCl_2_·2H_2_O (1.50 mM), 0.415 mg KI (2.50 μM), 3.150 mg H_3_BO_3_ (0.05 mM), 8.450 mg MnSO_4_·H_2_O (0.05 mM), 4.300 mg ZnSO_4_·7H_2_O (0.015 mM), 0.125 mg Na_2_MoO_4_·2H_2_O (0.50 μM), 0.0125 mg CuSO_4_·5H_2_O (0.05 μM), 0.0125 mg CoCl_2_·6H_2_O (0.05 μM), 18.65 mg Na_2_·EDTA (0.05 mM), 13.90 mg FeSO_4_·7H_2_O (0.05 mM), 51.55 mg vitamin (Duchefa, Haarlem, The Netherlands), 15 g sucrose, and 8 g agar. The pH of the medium was 5.8. The nitrogen deficient medium did not contain NH_4_NO_3_ and KNO_3_; instead 0.950 g KCl was added for providing K. For germination, seeds sown in two different nutrient conditions were incubated for 48 h at 25 °C in the dark. After germination, the sprouts were incubated under a 16-h light/8-h dark photoperiod and light intensity of approximately 4000 lx at 25 °C. The samples were harvested at six different times, immediately after incubation in the dark (0 DAI), and at 1, 2, 3, 5, and 7 DAI. The harvested samples were freeze-dried for six days, powdered, and stored at −80 °C until analysis.

### 3.2. Extraction of Hydrophilic Metabolites and GC-TOFMS Analysis

The freeze-dried radish sprouts (10 mg) and 1 mL of 2.5:1:1 (*v/v*) methanol:water:chloroform were blended with 60 µL of ribitol (adonitol, 200 μg/mL), which was used as an internal standard (IS). The mixture was shaken at 1,200 rpm for 30 min at 37 °C using a Thermomixer Comfort (model 5355, Eppendorf AG, Hamburg, Germany). The mixture was centrifuged at 16,000× *g* for 3 min at 4 °C. The aqueous supernatant (800 µL) obtained after centrifugation was mixed with 400 µL of deionized water. After vortexing, the mixture was centrifuged at 16,000× *g* for 3 min at 4 °C. The supernatant (900 µL), thus obtained, was completely dried using a vacuum centrifuge dryer (CC-105, TOMY, Tokyo, Japan) and freeze-dried (MCFD8512, IlShinBioBase, Dongducheon, Korea). Thereafter, 80 µL of methoxamine (MOX) reagent (Thermo Fisher Scientific, Waltham, MA, USA) was added to the concentrated sample and incubated at 30 °C, with shaking at 1200 rpm for 90 min. This was followed by addition of 80 µL of *N*-methyl-*N*-(trimethylsilyl) trifluoroacetamide (MSTFA; Sigma, St. Louis, MO, USA) and incubation (with shaking at 1200 rpm) at 37 °C for 30 min. The separation of hydrophilic compounds was carried out using a CP-SIL 8 CB-MS column (30 m × 0.25 mm, 0.25 mm, Agilent, Massy, France) on an Agilent 6890N Network GC system (Agilent) coupled to a Pegasus HT TOF mass spectrometer (LECO, St Joseph, MI, USA). The derivatized sample (1 μL) was injected with a split ratio of 1:25 at 230 °C and helium gas was passed at a flow rate of 1 mL/min. The column temperature was maintained at 80 °C for 2 min; it was increased at 15 °C/min to 320 °C and held at this temperature for 10 min. The ion source and transfer line temperature were set at 200 °C and 250 °C, respectively. The mass voltage was set at 1700 volt and the mass range for scanning was 85 to 600. The qualitative analysis was performed by comparison with the respective standards, possessed in an in-house library [52]. The quantitative estimation was based on peak area ratios relative to the IS peak area.

### 3.3. Extraction of Policosanols, Tocopherols, and Phytosterols and GC-qMS Analysis

The extraction of policosanols, tocopherols, and phytosterols was performed using previously described methods [53]. The powdered sample (50 mg) was vortexed with 3 mL of ethanol containing 0.1% (*w/v*) ascorbic acid and 10 μg/mL 5α-cholestane as an IS. After incubation at 85 °C for 5 min, for saponification, 120 μL of 80% (*w/v*) KOH was added and the mixture was incubated at 85 °C for 10 min. The saponified sample was chilled on ice for 5 min, mixed with 1.5 mL hexane and deionized water, vortexed, and centrifuged at 1200× *g* for 5 min at 4 °C. The hexane layer was collected in a new tube and re-extracted with an equal volume of hexane. The collected hexane layer was concentrated using nitrogen gas. For derivatization, 30 μL of MSTFA and pyridine were added and incubated at 60 °C, with shaking at 1,200 rpm for 30 min. Thereafter, 1 μL of each derivatized sample was injected into an Rtx-5MS column (30 m × 0.25 mm, 0.25 μm; Restek, Bellefonte, PA, USA) attached to a GC-qMS instrument (GCMS-QP2010 Ultra system; Shimadzu, Kyoto, Japan). The injection temperature and split ratio were 290 °C and 10:1, respectively. The oven temperature was initially kept at 150 °C for 2 min; it was then increased at a rate of 15 °C/min to 320 °C, and maintained at this temperature for 10 min. The carrier gas, helium, was passed at a flow rate of 1 mL/min. The ion source and interface temperature were set at 230 and 280 °C, respectively. The qualitative and quantitative analyses of lipophilic compounds were performed as described by Kim et al. (2015) [53].

### 3.4. Extraction of Carotenoids and HPLC Analysis

The method for extraction and analysis was as described by Park et al. (2014) [54]. For the extraction of carotenoids, 10–100 mg of sample was mixed with 3 mL ethanol (containing 0.1% (*w/v*) ascorbic acid). The mixture was vortexed and incubated in a water bath at 85 °C for 5 min. For saponification, 120 μL of 80% (*w/v*) KOH was added and the mixture was incubated at 85 °C for 10 min and cooled on ice. This was followed by addition of 1.5 mL of hexane and deionized water. As an IS, *β*-apo-8′-carotenal (100 μL, 25 μg/mL) was used. The mixture was centrifuged at 1200× *g* for 5 min at 4 °C and the hexane layer was transferred into a new tube. After repeating the process of adding and collecting the hexane layer, the collected hexane fractions were dried under nitrogen gas. Thereafter, 250 μL of 50:50 (*v/v*) dichloromethane:methanol was used as the solvent. For analysis, an Agilent 1100 series HPLC instrument (Agilent), equipped with YMC Carotenoid S-3 μm column (250 × 4.6 mm, 3 μm; YMC Co., Kyoto, Japan) and photodiode array detector, was used. The chromatographic signal was measured at 450 and 286 nm. A gradient elution was performed using 92:8 (*v/v*) methanol:water with 10 mM ammonium acetate (solvent A) and methyl tert-butyl ether (solvent B) at a flow rate of 1 mL/min. The gradient used for elution was as follows: 0 min, 90% A/10% B; 20 min, 83% A/17% B; 29 min, 75% A/25% B; 35 min, 30% A/70% B; 40 min, 30% A/70% B; 42 min, 25% A/75% B; 45 min, 90% A/10% B; 55 min, 90% A/10% B. The column temperature was set at 40 °C. The qualitative and quantitative analyses of carotenoids were conducted using calibration curves made for the standard compounds.

### 3.5. Extraction of Chlorophylls and Analysis

Total chlorophylls were extracted from 10–30 mg of samples using 100% methanol at 70 °C for 30 min with Thermomixer Comfort (Eppendorf AG) at 500 rpm speed. The sample was centrifuged at 4 °C and 3000 rpm for 10 min, and the absorbance of the supernatant was measured at 666 and 653 nm to calculate the chlorophyll content using the formula mentioned by Wellburn (1994) [55].

### 3.6. Extraction of Desulfoglucosinolates and HPLC Analysis

To 100 mg of sample, we added boiling 70% (*v/v*) methanol (1.5 mL) and incubated at 69 °C. The mixture was centrifuged at, 13,000× *g* for 10 min at 4 °C. The supernatant was collected, and the extraction step was repeated two more times. The collected supernatant (4.5 mL) was loaded onto a prepared disposable chromatography column (Bio-Rad Laboratories Hercules, CA, USA), which was filled with DEAE Sephadex A-25 (GE Healthcare, Uppsala, Sweden) using 0.5 M sodium acetate. As an external standard (ES), 200 μL of 2.5 mM sinigrin was loaded onto another column at the same time. After washing with 3 mL of deionized water, 70 µL of purified sulfatase (Sigma) was added to the column and incubated at 25 °C for 16 h. The desulfoglucosinolates were eluted with 2.4 mL of deionized water. The eluate, passed through the column, was filtered with a PTFE (0.20 µm) hydrophilic syringe filter (Advantec, Tokyo, Japan) for analysis. The sample (20 µL) was injected and separated on a C18 column (250 × 4.6 mm, 5 µm, Inertsil ODS-3; GL Sciences, Tokyo, Japan) using a Waters HPLC (e2695; Milford, MA, USA). The chromatograms were generated at 227 nm using a Waters 2998 photodiode array detector. The third distilled water and acetonitrile were used as gradient elution solvents A and B, respectively. The gradient program used was as follows: 0 min, 99% A/1% B; 18 min, 80% A/20% B; 30 min, 80% A/20% B; 35 min, 70% A/30% B; 37 min, 99% A/1% B; 47 min, 99% A/1% B. The solvent flow was 1 mL/min and column temperature was set at 40 °C. Peak identification was done using the procedure described by Baek et al. (2016) [17]. The content of glucosinolates was calculated using the response factor of each compound relative to that of sinigrin [56].

### 3.7. Statistical Analysis

All the experiments, except for phenotypic feature measurements (n = 6), were carried out in biological triplicates. The normalization (unit variance (UV) scaling) of metabolite data and PCA were performed using SIMCA (version 14.1, Umetrics, Umea, Sweden). The results of Student’s *t*-test were obtained using the SAS 9.4 software (SAS Institute, Cary, NC). MetaboAnalyst 4.0 (http://www.metaboanalyst.ca) was used for preparing the heat map. PathVisio 3.3.0 was downloaded from the PathVisio website (https://www.pathvisio.org) and used for visualization of the metabolite changes in metabolites.

## 4. Conclusions

In this study, we performed metabolite profiling of radish sprouts under N sufficient and –N conditions. For the first time, we used PathVisio to present the metabolic changes in pathway diagrams in response to –N conditions in radish sprouts. To the best of our knowledge, our results show for the first time that comprehensive metabolic profiling of radish sprouts reveals rapid response after germination to –N conditions. We found that not only was there a change in the contents of metabolites with growth of sprouts, but also there was a difference in metabolite flow between N sufficient and –N conditions during the development, from germination to the formation of true leaves. Monosaccharides, pigments, and α-, β-tocopherols increased with plant growth. Low levels of amino acids and organic acids were detected in radish sprouts grown under –N conditions. On the other hand, accumulation of sugars and γ-tocopherol was detected in radish sprouts grown under –N conditions. The dramatic change in the contents of amino acids occurred within 1 DAI, even at 0 DAI (dark incubation period for germination). This means that radish seeds could recognize the surrounding nutrient situation and reacted quickly to adapt to or overcome it. An important issue for future work is to investigate the effects of N starvation on the levels of metabolites and expression of genes in the radish sprouts. Thus, future work will focus on obtaining integrated omics data from radish sprouts grown under –N conditions for short (within 1 DAI) periods. Our results provide information about the changes in the levels of metabolites during plant developmental processes and about the system of usage of N. In addition, it offers information that would help in understanding the recognition and response mechanism against N deficiency in plants.

## Supplementary Materials

The following are available online at https://www.mdpi.com/2223-7747/8/10/361/s1, Figure S1: GC-TOFMS chromatogram of hydrophilic compounds in radish sprouts (0 DAI) grown under nitrogen sufficient condition; Figure S2: HPLC chromatogram of carotenoids in radish sprouts (0 DAI) grown under nitrogen sufficient condition; Figure S3: HPLC chromatogram of glucosinolates in radish sprouts (0 DAI) grown under nitrogen sufficient condition; Figure S4: Metabolite expression data mapped onto the schematic pathway of radish sprouts grown under (A) nitrogen sufficient and (B) nitrogen deficient conditions; Table S1: Phenotypic changes in radish sprouts during nitrogen deficient condition; Table S2: Composition and abundance of hydrophilic compounds in radish sprouts grown under nitrogen sufficient condition; Table S3: Composition and abundance of hydrophilic compounds in radish sprouts grown under nitrogen deficient condition; Table S4: Composition and abundance of policosanols, tocopherols, and phytosterols in radish sprouts grown under nitrogen sufficient condition; Table S5: Composition and abundance of policosanols, tocopherols, and phytosterols in radish sprouts grown under nitrogen deficient condition; Table S6: Composition and abundance of pigments in radish sprouts grown under nitrogen sufficient condition; Table S7: Composition and abundance of pigments in radish sprouts grown under nitrogen deficient condition; Table S8: Composition and abundance of glucosinolates in radish sprouts grown under nitrogen sufficient condition; Table S9: Composition and abundance of glucosinolates in radish sprouts grown under nitrogen deficient condition.
