# Supplementary material for: Dynamics of Short-Term Metabolic Profiling in Radish Sprouts (Raphanus sativus L.) in Response to Nitrogen Deficiency
Source: Plants (Basel). 2019 Sep 23;8(10):361. doi: 10.3390/plants8100361 (PMC6843509; doi:10.3390/plants8100361)

Table S1. Phenotypic changes in radish sprouts during nitrogen deficient condition. Fresh weight, dry weight, length of shoot and root of radish sprouts grown under the two nitrogen conditions.

Phenotype[a]		0 DAI[b]	1 DAI	2 DAI	3 DAI	5 DAI	7 DAI
Nitrogen sufficient condition							
Weight (mg)	Fresh sample	58.97±12.67	139.95±34.28	**225.05±19.31**	244.53±27.32	288.63±81.01	**358.05±82.25**
	Dry sample	15.05±1.49	17.30±4.54	18.20±2.11	16.10±2.92	16.02±2.58	20.30±3.61
Length (mm)	Shoot	20.64±7.28	**33.71±7.10**[c]	50.41±4.60	71.39±13.28	70.58±11.09	80.46±15.62
	Root	11.62±2.42	49.59±11.10	**61.57±4.12**	60.26±14.42	62.94±13.48	**60.72±9.73**
Nitrogen deficient condition							
Weight (mg)	Fresh sample	57.65±12.54	112.10±29.63	**148.37±18.94**	204.93±53.15	225.47±47.17	**257.27±64.31**
	Dry sample	13.13±1.89	14.32±2.56	18.20±5.18	18.70±5.43	16.57±2.55	18.13±5.05
Length (mm)	Shoot	24.00±3.09	**25.12±4.29**	44.74±5.69	57.90±9.56	70.59±11.82	73.22±8.89
	Root	9.03±1.52	53.73±17.80	**76.05±8.51**	68.22±14.70	63.08±13.18	**84.63±6.89**

[a] Each value is the mean of six replications ± standard deviation. [b] Day after light incubation began. [c] Statistically significant mean scores ($p \leq 0.05$) are highlighted in **bold**.

Table S2. Composition and abundance of hydrophilic compounds (ratio/g of dry weight) in radish sprouts grown under nitrogen sufficient condition.

Compound[a]	0 DAI[b]	1 DAI	2 DAI	3 DAI	5 DAI	7 DAI
Organic acids and inorganic acid						
Citric acid	13.83±1.54	**22.34±2.90**	**13.91±1.57**	**9.61±1.09**	**10.01±1.94**	**9.75±1.31**
Ferulic acid	1.44±0.34	1.19±0.10	**1.09±0.16**	**1.29±0.15**	1.08±0.20	1.27±0.39
Fumaric acid	3.08±0.66	**5.28±0.37**	**6.13±0.62**	**7.71±1.29**	**6.15±0.26**	6.09±1.71
Glyceric acid	**6.42±3.39**[c]	**6.38±0.54**	**8.05±2.49**	**5.44±1.30**	5.02±1.35	**8.51±0.98**
Lactic acid	4.90±0.69	7.51±3.80	11.48±4.52	**11.83±2.74**	14.67±2.37	34.56±3.11
Malic acid	56.40±2.58	**141.87±11.59**	**165.32±7.18**	**185.59±5.69**	**172.92±25.35**	**306.98±58.05**
Pyruvic acid	0.37±0.10	0.87±0.18	0.98±0.06	1.12±0.18	**0.85±0.12**	1.00±0.33
Sinapic acid	20.10±3.94	**20.09±0.30**	**16.97±0.51**	22.86±4.37	17.20±2.86	**18.72±2.97**
Succinic acid	**20.90±3.10**	**82.14±12.88**	**37.35±2.19**	**31.03±3.21**	**20.96±2.16**	**22.58±1.95**
Threonic acid	1.23±0.25	4.46±1.16	3.97±1.31	**3.77±0.60**	**3.24±0.71**	**4.58±1.02**
Phosphoric acid	80.98±6.63	**205.33±9.34**	310.05±14.47	**330.38±27.76**	**429.32±39.88**	508.32±67.08
Amino acids						
Alanine	212.19±61.20	173.88±57.62	**226.31±62.81**	**253.87±72.93**	**325.92±87.54**	**373.19±76.50**
β-Alanine	2.50±0.23	4.31±1.05	**5.44±0.24**	**3.73±0.58**	**3.18±0.12**	**2.59±0.40**
4-Aminobutanoic acid	7.90±1.67	11.07±3.09	**25.22±4.19**	**23.36±3.75**	**31.31±5.05**	**30.22±2.71**
Asparagine	10.21±3.12	11.66±2.53	**24.25±4.22**	**46.46±4.50**	**61.62±9.08**	**91.39±26.68**
Aspartic acid	25.54±2.07	**23.59±0.88**	**26.65±4.39**	**34.22±8.34**	**29.25±7.81**	**41.41±7.66**
Cysteine	3.77±0.90	3.28±0.89	**4.12±0.41**	4.20±0.45	**5.70±0.60**	4.96±1.14
Glutamic acid	111.51±17.90	107.70±24.38	**82.38±7.45**	**69.89±9.48**	**78.55±3.23**	**79.26±15.21**
Glutamine	12.52±5.89	10.45±7.02	16.58±9.23	24.20±11.26	**27.85±5.52**	59.43±38.88
Glycine	**57.17±13.26**	89.45±20.90	**121.25±17.57**	**98.70±22.78**	**116.44±19.83**	**96.35±16.78**
Isoleucine	**23.17±3.48**	**29.75±13.60**	45.89±10.20	49.59±6.31	**105.03±6.29**	96.18±7.30
Leucine	**28.71±3.81**	**23.59±12.97**	31.96±9.22	30.79±2.35	**45.18±3.01**	**31.48±3.37**
Lysine	**4.21±1.09**	**6.12±2.67**	14.93±3.36	28.14±4.51	**60.83±0.48**	56.27±3.46
Methionine	**2.73±0.18**	**3.13±0.68**	**5.80±0.41**	9.01±0.98	**17.44±0.34**	17.02±5.47
Phenylalanine	**7.30±0.78**	**5.68±0.97**	**6.03±0.90**	8.97±1.08	**28.96±1.18**	**29.55±3.67**

Table S2. (*Continued*)

Compound	0 DAI	1 DAI	2 DAI	3 DAI	5 DAI	7 DAI
Amino acids						
Proline	242.53±57.4	**139.10±84.42**	**138.05±55.12**	95.31±15.23	84.30±30.36	**75.14±10.54**
Pyroglutamic acid	162.64±10.79	**238.06±38.69**	**375.36±44.86**	**527.31±36.43**	**697.44±167.14**	667.16±300.08
Serine	11.74±1.13	22.64±17.19	19.79±2.87	**32.37±9.52**	**30.84±8.80**	**40.02±12.64**
Threonine	**16.48±1.37**	17.29±4.57	**25.94±3.28**	**32.50±1.42**	**61.19±3.56**	**74.17±3.53**
Tryptophan	**6.50±1.50**	**4.01±1.86**	3.32±0.54	**2.90±0.48**	10.95±2.00	12.12±1.01
Valine	**62.80±7.62**	70.26±20	**92.79±19.31**	99.50±15.53	**211.26±21.42**	**201.71±16.77**
Sugars and sugar alcohols						
Arabinose	4.30±1.21	**9.06±0.27**	**12.27±0.49**	13.10±1.04	10.01±2.32	8.05±1.66
Fructose	2176.61±673.53	4856.54±1094.66	6000.16±1429.11	5111.44±1403.31	5561.79±1675.52	4192.56±1564.09
Galactose	1.26±0.35	20.64±15.59	53.97±12.06	42.73±13.12	19.82±13.09	**5.39±3.43**
Glucose	571.84±233.22	1583.26±547.69	2023.47±760.05	1353.21±768.43	1467.01±664.84	1377.54±1255.09
Raffinose	2.61±0.48	2.25±0.25	1.85±0.23	1.24±0.24	1.35±0.19	1.40±0.39
Sucrose	643.26±114.08	531.08±80.86	511.02±88.72	414.15±33.91	432.67±86.03	**418.41±46.50**
Xylose	30.31±8.11	67.84±5.88	**107.12±12.51**	**148.93±14.43**	172.64±44.22	**162.59±33.38**
Glycerol	18.69±3.26	29.97±2.97	50.15±5.86	56.49±8.64	61.47±11.88	102.28±11.09
Inositol	88.38±15.49	150.46±18.85	134.49±17.31	105.64±12.26	91.37±6.06	72.33±1.32
Amines and amide						
Ethanolamine	22.75±5.44	10.57±4.10	14.14±1.73	15.54±5.59	**21.28±1.94**	**31.02±5.23**
Putrescine	**24.97±3.03**	45.53±16.92	**89.94±12.29**	**90.32±11.54**	156.36±23.03	**187.26±39.06**
Urea	0.26±0.06	0.34±0.09	**0.98±0.30**	1.51±0.13	**3.38±0.41**	**4.84±0.64**
Sugar phosphates						
Fructose 6-phosphate	1.57±0.52	3.30±0.56	3.56±0.38	2.31±0.50	2.28±0.81	1.89±0.92
Glucose 6-phosphate	3.57±1.15	7.68±1.19	7.82±1.17	4.65±1.53	4.23±1.86	3.09±1.44

[a] Each value is the mean of three replications ± standard deviation. [b] Day after light incubation began. [c] Statistically significant mean scores ($p \leq 0.05$) are highlighted in **bold**.

Table S3. Composition and abundance of hydrophilic compounds (ratio/g of dry weight) in radish sprouts grown under nitrogen deficient condition.

Compound[a]	0 DAI[b]	1 DAI	2 DAI	3 DAI	5 DAI	7 DAI
Organic acids and inorganic acid						
Citric acid	12.46±1.00	**11.06±0.62**	**4.21±0.80**	**3.92±0.98**	**3.71±0.52**	**3.71±0.47**
Ferulic acid	1.41±0.28	1.07±0.14	**0.75±0.14**	**0.84±0.13**	**0.77±0.10**	0.75±0.11
Fumaric acid	2.01±0.30	**2.42±0.40**	**2.61±0.42**	**3.13±0.52**	2.76±0.43	2.40±0.23
Glyceric acid	**47.66±5.83**[c]	**59.00±8.42**	**42.85±10.39**	**28.69±4.98**	6.01±4.13	**1.06±0.15**
Lactic acid	5.93±1.88	8.81±4.62	9.78±1.25	**18.64±1.36**	11.66±4.64	28.00±3.64
Malic acid	63.59±9.07	**63.28±2.90**	**51.61±8.28**	**39.85±4.44**	**27.04±2.64**	**18.58±2.34**
Pyruvic acid	0.42±0.05	0.81±0.14	1.13±0.19	0.84±0.10	**0.61±0.04**	0.85±0.06
Sinapic acid	20.75±1.35	**17.15±1.07**	**13.96±1.50**	16.35±3.95	12.72±0.41	**11.61±0.74**
Succinic acid	**11.70±0.80**	**20.42±3.70**	**9.54±1.21**	**4.42±0.24**	**2.95±0.50**	**2.62±0.29**
Threonic acid	1.30±0.12	2.90±0.64	2.96±0.22	**2.16±0.16**	**1.71±0.30**	**1.63±0.26**
Phosphoric acid	86.53±2.13	**185.73±7.70**	340.57±51.79	**419.09±34.61**	**596.81±36.23**	605.24±61.68
Amino acids						
Alanine	169.72±25.47	109.75±12.60	**36.22±16.05**	**63.51±11.39**	**76.25±20.61**	**60.13±13.11**
β-Alanine	2.44±0.19	3.21±0.05	**1.84±0.56**	**1.58±0.06**	**1.29±0.08**	**1.11±0.06**
4-Aminobutanoic acid	9.22±1.21	11.22±0.52	**10.43±3.48**	**8.62±1.43**	**5.20±0.41**	**7.59±0.68**
Asparagine	7.20±1.42	8.20±2.30	**4.63±1.84**	**12.05±0.54**	**21.64±1.68**	**27.56±4.70**
Aspartic acid	25.30±3.11	**17.10±3.17**	**10.65±2.46**	**11.10±1.49**	**8.44±1.11**	**6.88±1.73**
Cysteine	5.79±0.98	3.79±0.28	**2.39±0.42**	3.56±0.30	**4.30±0.52**	4.42±0.43
Glutamic acid	115.05±11.93	75.98±6.69	**25.46±4.99**	**30.53±7.53**	**31.74±4.19**	**27.98±2.44**
Glutamine	6.99±2.24	6.83±1.51	1.87±1.29	8.79±1.71	**13.97±3.29**	16.77±1.76
Glycine	**34.17±3.67**	42.10±2.72	**21.12±6.28**	**27.39±2.02**	**30.61±5.83**	**24.11±4.41**
Isoleucine	**35.60±2.09**	**55.72±5.14**	32.92±10.41	60.64±3.56	**82.01±8.17**	80.4±11.28
Leucine	**51.93±2.35**	**49.17±5.75**	17.74±6.94	30.04±3.53	**23.67±2.35**	**20.26±2.53**
Lysine	**7.71±0.96**	**10.90±0.89**	11.63±3.33	34.20±2.80	**47.31±4.89**	51.72±4.88
Methionine	**5.29±0.41**	**5.19±0.50**	**3.96±1.02**	8.04±0.14	**11.78±0.84**	14.69±0.90
Phenylalanine	**15.52±1.19**	**9.35±0.46**	**4.33±1.19**	8.35±0.85	**16.50±1.67**	**21.77±2.69**

Table S3. (*Continued*)

Compound	0 DAI	1 DAI	2 DAI	3 DAI	5 DAI	7 DAI
Amino acids						
Proline	342.38±68.49	**305.50±56.12**	**46.05±12.96**	105.17±26.50	61.67±8.36	**30.74±10.70**
Pyroglutamic acid	122.98±23.60	**95.62±15.97**	**102.72±11.07**	**131.50±28.23**	**176.91±29.47**	224.64±80.37
Serine	13.48±1.47	12.07±2.88	22.33±9.42	**12.64±3.12**	**11.12±1.73**	**11.21±3.01**
Threonine	**22.71±0.68**	24.26±1.28	**15.70±3.76**	**27.98±2.24**	**43.27±2.82**	**52.70±3.37**
Tryptophan	**10.56±0.61**	**7.66±1.15**	2.20±1.24	**6.29±1.70**	11.69±2.35	14.63±2.55
Valine	**82.68±5.68**	92.57±8.22	**50.59±17.58**	89.34±5.32	**125.73±11.36**	**121.53±17.53**
Sugars and sugar alcohols						
Arabinose	4.60±0.25	**8.32±0.29**	**11.22±0.36**	11.26±1.40	10.19±0.68	9.43±0.51
Fructose	2873.19±239.12	5294.15±991.95	4996.05±97.42	6395.74±1750.43	7779.05±1501.21	7581.89±1556.63
Galactose	2.06±0.62	5.78±0.67	28.25±13.29	58.36±4.65	101.77±60.78	**64.96±4.53**
Glucose	768.07±144.05	1760.39±571.21	1307.50±41.51	1884.07±978.01	2272.34±765.70	2234.27±744.32
Raffinose	2.84±0.19	2.29±0.24	1.42±0.48	1.80±0.43	1.69±0.25	1.60±0.19
Sucrose	802.66±93.12	757.66±144.11	529.83±26.24	585.23±137.67	625.55±85.47	**668.22±108.25**
Xylose	34.23±2.02	61.17±2.15	**84.46±5.69**	**89.56±11.70**	88.94±2.78	**83.78±9.87**
Glycerol	21.89±2.60	32.51±6.32	44.01±3.71	64.20±10.14	68.78±9.54	92.52±2.31
Inositol	73.77±8.67	156.04±21.21	136.28±2.10	130.44±20.10	101.58±17.72	91.92±14.06
Amines and amide						
Ethanolamine	23.35±0.91	16.75±3.79	8.63±3.35	13.90±0.46	**11.44±1.57**	**11.72±1.25**
Putrescine	**14.87±1.00**	40.55±1.07	**53.24±15.94**	**117.80±10.68**	184.62±16.93	**270.25±17.67**
Urea	0.30±0.05	0.37±0.10	**0.37±0.09**	1.05±0.31	**0.66±0.11**	**1.61±0.32**
Sugar phosphates						
Fructose 6-phosphate	1.64±0.28	2.94±0.32	2.55±0.62	2.76±0.46	2.19±0.29	2.15±0.28
Glucose 6-phosphate	3.88±0.74	6.93±0.73	5.43±1.47	6.09±1.12	4.64±0.78	3.98±0.30

[a] Each value is the mean of three replications ± standard deviation. [b] Day after light incubation began. [c] Statistically significant mean scores ($p \leq 0.05$) are highlighted in **bold**.

Table S4. Composition and abundance of policosanols, tocopherols and phytosterols (μg/g of dry weight) in radish sprouts grown under nitrogen sufficient condition.

Compound[a]	0 DAI[b]	1 DAI	2 DAI	3 DAI	5 DAI	7 DAI
Policosanols						
C20	4.57±0.52	4.44±0.53	**3.77±0.54**[c]	**4.64±0.17**	6.58±0.55	8.82±1.58
C21	1.76±0.07	1.59±0.60	1.76±0.15	1.96±0.29	2.16±0.20	2.19±0.29
C22	76.94±11.78	66.44±9.41	57.54±15.49	76.68±4.22	74.35±11.16	83.29±4.54
C23	5.67±0.55	5.55±0.97	4.14±0.75	3.59±1.39	4.35±1.28	4.63±0.38
C24	26.37±3.41	37.75±12.86	40.36±10.94	49.17±11.30	56.36±15.67	53.92±10.96
C26	5.14±0.42	9.03±4.24	20.59±10.84	36.23±8.89	40.00±11.02	32.24±10.08
C27	2.87±0.13	3.50±0.58	4.80±1.24	6.40±1.47	7.07±1.02	7.37±0.80
C28	10.50±1.41	14.27±2.11	20.16±2.87	**37.06±2.78**	50.29±16.82	60.15±34.33
C30	11.49±2.84	16.07±0.90	16.78±5.26	23.59±6.10	21.80±1.96	24.88±2.18
Total	145.31±21.14	158.64±32.19	169.90±48.08	239.32±36.62	262.98±59.68	277.50±65.15
Tocopherols						
α-Tocopherol	26.56±1.13	90.50±3.86	151.53±14.54	192.48±16.27	216.73±22.59	248.11±33.75
β-Tocopherol	2.06±0.14	3.11±0.30	4.47±0.27	5.11±0.42	5.06±0.66	5.57±0.88
γ-Tocopherol	114.02±12.83	71.10±5.92	**40.09±0.93**	**20.32±2.19**	**9.37±2.78**	5.48±1.14
Total	142.64±14.1	164.72±10.08	196.09±15.74	217.91±18.88	231.16±26.04	259.16±35.77
Phytosterols						
Brassicasterol	203.94±4.44	185.16±15.06	165.69±10.90	**139.18±8.71**	115.72±10.78	99.31±14.84
Campesterol	856.50±39.31	810.60±99.65	873.39±12.13	907.65±54.09	912.20±49.78	921.79±43.89
Cholesterol	36.62±2.36	35.32±5.07	37.53±1.87	41.41±4.31	40.05±3.90	38.81±1.49
β-Sitosterol	820.90±48.23	816.37±91.85	895.4±50.85	982.22±79.06	932.48±50.41	1030.24±82.66
Stigmasterol	11.66±2.28	38.97±6.83	62.78±7.01	81.39±8.64	90.39±21.45	116.82±9.67
Total	1929.61±96.62	1886.42±218.46	2034.79±82.76	2151.85±154.82	2090.84±136.32	2206.96±152.54

[a] C20, eicosanol; C21, heneicosanol; C22, docosanol; C23, tricosanol; C24, tetracosanol; C26, hexacosanol; C27, heptacosanol; C28, octacosanol; C30, triacontanol. Each value is the mean of three replications ± standard deviation. [b] Day after light incubation began. [c] Statistically significant mean scores ($p \leq 0.05$) are highlighted in **bold**.

Table S5. Composition and abundance of policosanols, tocopherols and phytosterols (μg/g of dry weight) in radish sprouts grown under nitrogen deficient condition.

Compound[a]	0 DAI[b]	1 DAI	2 DAI	3 DAI	5 DAI	7 DAI
Policosanols						
C20	4.46±0.33	4.54±0.18	**5.09±0.59**[c]	**6.18±0.21**	6.63±0.10	9.98±2.41
C21	1.86±0.11	1.60±0.22	2.11±0.36	1.92±0.18	2.14±0.27	1.83±0.13
C22	68.65±33.66	73.23±9.69	76.58±7.62	72.07±0.69	79.26±5.86	66.81±14.58
C23	6.55±0.83	5.09±2.01	4.20±1.31	4.07±0.91	3.59±1.70	5.14±0.63
C24	25.48±9.12	37.64±9.60	45.39±13.66	51.43±6.65	59.39±14.25	66.77±6.29
C26	5.08±1.37	7.61±2.34	16.39±5.67	22.25±6.60	25.13±7.12	31.08±14.03
C27	2.84±0.23	3.30±0.24	4.21±0.57	4.45±0.55	5.81±0.72	8.22±0.92
C28	8.98±1.44	14.61±2.59	18.13±3.28	**24.49±3.12**	34.82±8.89	45.80±15.16
C30	13.48±1.38	17.60±4.24	23.54±4.57	26.54±2.07	20.69±1.73	24.00±3.76
Total	137.39±48.48	165.22±31.11	195.63±37.64	213.40±20.98	237.46±40.64	259.63±57.92
Tocopherols						
α-Tocopherol	25.04±2.35	98.06±10.71	147.04±10.09	179.62±11.63	229.69±24.43	287.99±42.07
β-Tocopherol	2.18±0.01	3.15±0.12	4.00±0.29	4.98±0.23	6.28±1.12	6.85±0.71
γ-Tocopherol	107.90±5.80	73.43±6.09	**50.47±3.60**	**31.92±1.00**	**16.50±2.81**	8.05±2.05
Total	135.13±8.16	174.64±16.92	201.51±13.98	216.52±12.86	252.47±28.35	302.89±44.82
Phytosterols						
Brassicasterol	194.67±17.03	190.36±13.35	175.61±14.39	**154.57±3.80**	132.67±16.12	123.38±18.77
Campesterol	866.65±58.06	821.00±85.82	853.67±112.42	899.48±19.46	919.83±51.63	967.70±108.24
Cholesterol	32.13±1.64	31.90±7.44	35.14±2.18	38.79±2.27	37.02±4.23	41.11±6.44
β-Sitosterol	797.02±72.75	849.62±134.80	885.59±140.50	938.33±51.47	945.26±99.42	1022.55±120.46
Stigmasterol	12.85±4.49	40.01±9.92	72.25±10.02	82.26±10.82	106.88±11.56	130.87±9.34
Total	1903.32±153.97	1932.88±251.33	2022.25±279.51	2113.42±87.83	2141.64±182.97	2285.62±263.25

[a] C20, eicosanol; C21, heneicosanol; C22, docosanol; C23, tricosanol; C24, tetracosanol; C26, hexacosanol; C27, heptacosanol; C28, octacosanol; C30, triacontanol. Each value is the mean of three replications ± standard deviation. [b] Day after light incubation began. [c] Statistically significant mean scores ($p \leq 0.05$) are highlighted in **bold**.

Table S6. Composition and abundance of carotenoids (μg/g of dry weight) and chlorophylls (μg/mg of dry weight) in radish sprouts grown under nitrogen sufficient condition.

Compound[a]	0 DAI[b]	1 DAI	2 DAI	3 DAI	5 DAI	7 DAI
Carotenoids						
α-Carotene	0.39±0.09	**12.97±2.51**[c]	**12.46±1.77**	**12.21±0.42**	**11.57±1.91**	10.58±2.81
β-Carotene	5.18±0.63	**261.66±29.18**	**499.37±75.55**	**550.87±85.80**	**513.51±40.41**	**445.63±48.31**
9*Z*-β-Carotene	0.88±0.12	**48.52±5.53**	**101.42±14.67**	**104.75±15.41**	**101.90±2.52**	**100.45±20.46**
13*Z*-β-Carotene	1.15±0.14	**55.28±8.27**	**114.07±7.84**	**129.36±13.78**	**117.18±4.86**	103.93±17.41
β-Cryptoxanthin	0.80±0.19	**5.05±0.60**	3.70±1.17	3.47±0.95	2.46±0.85	1.78±0.67
Lutein	11.39±2.02	**266.97±34.75**	**417.77±58.51**	**448.57±75.19**	**453.76±48.97**	416.11±48.94
Violaxanthin	0.84±0.18	**7.85±1.85**	**13.18±1.91**	**16.13±2.02**	**18.07±1.63**	**18.08±1.82**
Zeaxanthin	0.26±0.08	**1.35±0.05**	ND[d]	ND	ND	ND
Total	20.89±3.46	659.65±82.74	1161.97±161.40	1265.36±193.57	1218.44±95.69	1096.56±139.33
Chlorophylls						
Chlorophyll *a*	0.009±0.001	**2.08±0.12**	**3.89±0.18**	**4.40±0.05**	**4.44±0.35**	**4.09±0.38**
Chlorophyll *b*	0.003±0.001	0.85±0.07	**1.46±0.08**	**1.73±0.03**	**1.82±0.25**	**1.59±0.19**
Total	0.013±0.001	2.93±0.19	5.35±0.26	6.13±0.08	6.27±0.60	5.68±0.56

[a] Each value is the mean of three replications ± standard deviation. [b] Day after light incubation began. [c] Statistically significant mean scores ($p \leq 0.05$) are highlighted in **bold**. [d] ND, not detectable.

Table S7. Composition and abundance of carotenoids (μg/g of dry weight) and chlorophylls (μg/mg of dry weight) in radish sprouts grown under nitrogen deficient condition.

Compound[a]	0 DAI[b]	1 DAI	2 DAI	3 DAI	5 DAI	7 DAI
Carotenoids						
α-Carotene	0.22±0.01	**6.85±1.24**[c]	**7.62±2.35**	**7.51±1.31**	**7.48±0.96**	6.86±0.37
β-Carotene	4.81±0.31	**189.64±14.96**	**330.75±50.01**	**358.90±16.55**	**381.72±52.47**	**349.99±25.06**
9*Z*-β-Carotene	0.77±0.09	**33.43±3.05**	**58.97±12.43**	**62.45±4.38**	**67.21±8.17**	**63.68±3.01**
13*Z*-β-Carotene	1.02±0.04	**38.04±6.38**	**74.25±8.72**	**82.15±4.39**	**84.70±13.25**	76.92±4.69
β-Cryptoxanthin	0.52±0.13	**3.00±0.31**	2.73±1.03	1.94±0.58	1.75±0.63	1.34±0.67
Lutein	9.82±0.89	**188.38±20.41**	**287.75±32.39**	**311.14±19.03**	**353.79±53.98**	342.89±25.25
Violaxanthin	0.65±0.08	**3.91±0.79**	**4.39±1.76**	**4.82±0.60**	**7.13±2.09**	**7.86±1.35**
Zeaxanthin	0.20±0.02	**0.90±0.04**	ND[d]	ND	ND	ND
Total	18.02±1.57	464.15±47.17	766.47±108.68	828.91±46.85	903.79±129.62	849.55±59.22
Chlorophylls						
Chlorophyll *a*	0.008±0.002	**1.58±0.22**	**2.75±0.14**	**3.25±0.25**	**3.11±0.28**	**3.29±0.26**
Chlorophyll *b*	0.006±0.002	0.63±0.15	**0.98±0.08**	**1.16±0.10**	**1.09±0.08**	**1.20±0.12**
Total	0.014±0.004	2.21±0.37	3.73±0.22	4.42±0.35	4.21±0.36	4.48±0.38

[a] Each value is the mean of three replications ± standard deviation. [b] Day after light incubation began. [c] Statistically significant mean scores ($p \leq 0.05$) are highlighted in **bold**. [d] ND, not detectable.

Table S8. Composition and abundance of aliphatic and indolic glucosinolates (μg/mg of dry weight) in radish sprouts grown under nitrogen sufficient condition.

Compound[a]	0 DAI[b]	1 DAI	2 DAI	3 DAI	5 DAI	7 DAI
Aliphatic glucosinolates						
Glucoalyssin	**0.48±0.05**[c]	0.19±0.03	0.11±0.04	0.11±0.03	0.09±0.04	0.07±0.02
Glucobrassicanapin	0.05±0.01	0.07±0.01	0.06±0.003	0.07±0.02	0.10±0.02	0.07±0.03
Glucoraphasatin	38.14±3.43	95.56±4.13	106.45±2.33	106.71±6.05	**111.59±5.32**	99.10±12.99
Progoitrin	0.30±0.04	0.40±0.05	0.52±0.05	0.60±0.16	0.54±0.09	0.44±0.15
Sinigrin	20.14±2.18	7.84±0.30	4.52±1.22	3.44±0.61	2.73±0.45	1.84±0.40
Total	59.11±5.72	104.07±4.52	111.65±3.64	110.93±6.88	115.04±5.92	101.51±13.58
Indolic glucosinolates						
4-Hydroxyglucobrassicin	2.05±0.06	1.06±0.52	1.15±0.26	**1.29±0.08**	0.74±0.11	0.56±0.23
Glucobrassicin	0.18±0.03	0.15±0.02	0.28±0.03	**0.34±0.04**	**0.48±0.06**	0.54±0.09
4-Methoxyglucobrassicin	0.08±0.01	0.22±0.04	**0.73±0.10**	**1.16±0.06**	**2.01±0.13**	**2.24±0.55**
Neoglucobrassicin	1.54±0.17	0.014±0.003	0.005±0.002	**0.008±0.0002**	**0.011±0.002**	0.013±0.004
Total	3.85±0.28	1.44±0.59	2.16±0.39	2.79±0.18	3.24±0.31	3.35±0.87

[a] Each value is the mean of three replications ± standard deviation. [b] Day after light incubation began. [c] Statistically significant mean scores ($p \leq 0.05$) are highlighted in **bold**.

Table S9. Composition and abundance of aliphatic and indolic glucosinolates (μg/mg of dry weight) in radish sprouts grown under nitrogen deficient condition.

Compound[a]	0 DAI[b]	1 DAI	2 DAI	3 DAI	5 DAI	7 DAI
Aliphatic glucosinolates						
Glucoalyssin	**0.60±0.04**[c]	0.20±0.05	0.13±0.08	0.10±0.03	0.07±0.03	0.06±0.01
Glucobrassicanapin	0.04±0.01	0.08±0.01	0.07±0.02	0.05±0.01	0.05±0.001	0.06±0.01
Glucoraphasatin	39.89±3.50	89.38±6.77	100.30±8.65	99.10±4.98	**84.33±5.79**	89.86±7.02
Progoitrin	0.31±0.04	0.42±0.10	0.50±0.16	0.45±0.03	0.43±0.07	0.41±0.10
Sinigrin	22.11±1.52	8.67±3.55	4.87±2.01	3.56±0.60	2.25±0.85	2.04±0.17
Total	62.95±5.11	98.75±10.48	105.87±10.92	103.27±5.65	87.13±6.74	92.42±7.31
Indolic glucosinolates						
4-Hydroxyglucobrassicin	2.23±0.21	1.17±0.66	0.75±0.25	**0.77±0.25**	0.55±0.12	0.51±0.14
Glucobrassicin	0.20±0.02	0.18±0.06	0.20±0.05	**0.24±0.01**	**0.32±0.07**	0.41±0.04
4-Methoxyglucobrassicin	0.08±0.01	0.20±0.08	**0.44±0.12**	**0.49±0.04**	**0.64±0.13**	**1.02±0.18**
Neoglucobrassicin	1.51±0.04	0.013±0.01	0.005±0.001	**0.004±0.001**	**0.004±0.001**	0.01±0.001
Total	4.02±0.28	1.56±0.81	1.39±0.43	1.51±0.29	1.51±0.32	1.94±0.37

[a] Each value is the mean of three replications ± standard deviation. [b] Day after light incubation began. [c] Statistically significant mean scores ($p \leq 0.05$) are highlighted in **bold**.

Figure S1. GC-TOFMS chromatogram of hydrophilic compounds in radish sprouts (0 DAI) grown under nitrogen sufficient condition. Peak:1, Pyruvic acid; 2, Lactic acid; 3, Alanine; 4, Valine; 5, Urea; 6, Serine-1; 7, Ethanolamine; 8, Phosphoric acid; 9, Glycerol; 10, Leucine; 11, Isoleucine; 12, Proline; 13, Glycine; 14, Succinic acid; 15, Glyceric acid; 16, Fumaric acid; 17, Serine-2; 18, Threonine; 19, β-Alanine; 20, Malic acid; 21, Aspartic acid; 22, Methionine; 23, Pyroglutamic acid; 24, 4-Aminobutyric acid; 25, Threonic acid; 26, Cysteine; 27, Glutamic acid; 28, Phenylalanine; 29, Xylose-1; 30, Xylose-2; 31, Arabinose; 32, Asparagine; 33, Adonitol (Internal standard); 34, Glutamine; 35, Putrescine; 36, Citric acid; 37, Fructose-1; 38, Fructose-2; 39, Galactose; 40, Glucose-1; 41, Glucose-2; 42, Lysine; 43, Inositol; 44, Ferulic acid; 45, Tryptophan; 46, Sinapinic acid; 47, Fructose-6-phosphate; 48, Glucose-6-phosphate-1; 49, Glucose-6-phosphate-2; 50, Sucrose; 51, Raffinose.

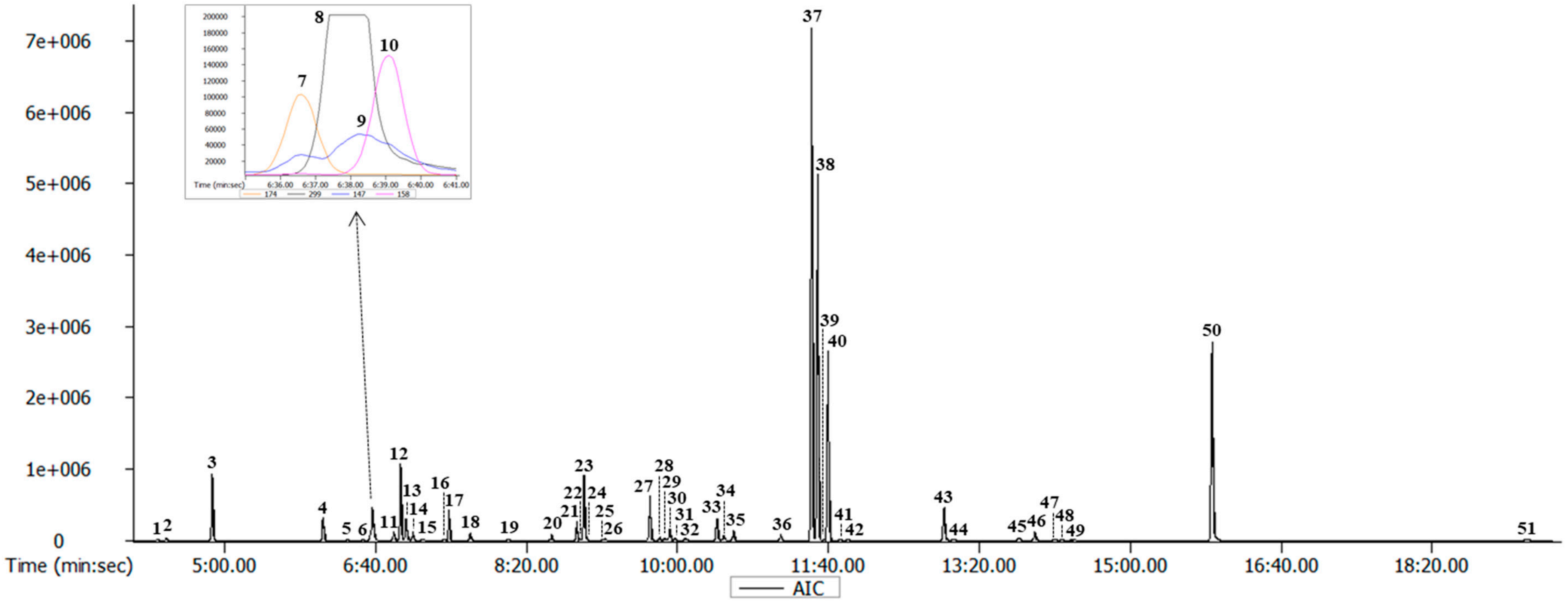

Figure S2. HPLC chromatogram of carotenoids in radish sprouts (0 DAI) grown under nitrogen sufficient condition. 1, Violaxanthin; 2, Lutein; 3, Zeaxanthin; 4, β-Apo-8’-carotenal (Internal standard); 5, β-Cryptoxanthin; 6, 13*Z*-β-Carotene; 7, α-Carotene; 8, β-Carotene; 9, 9*Z*-β-Carotene.

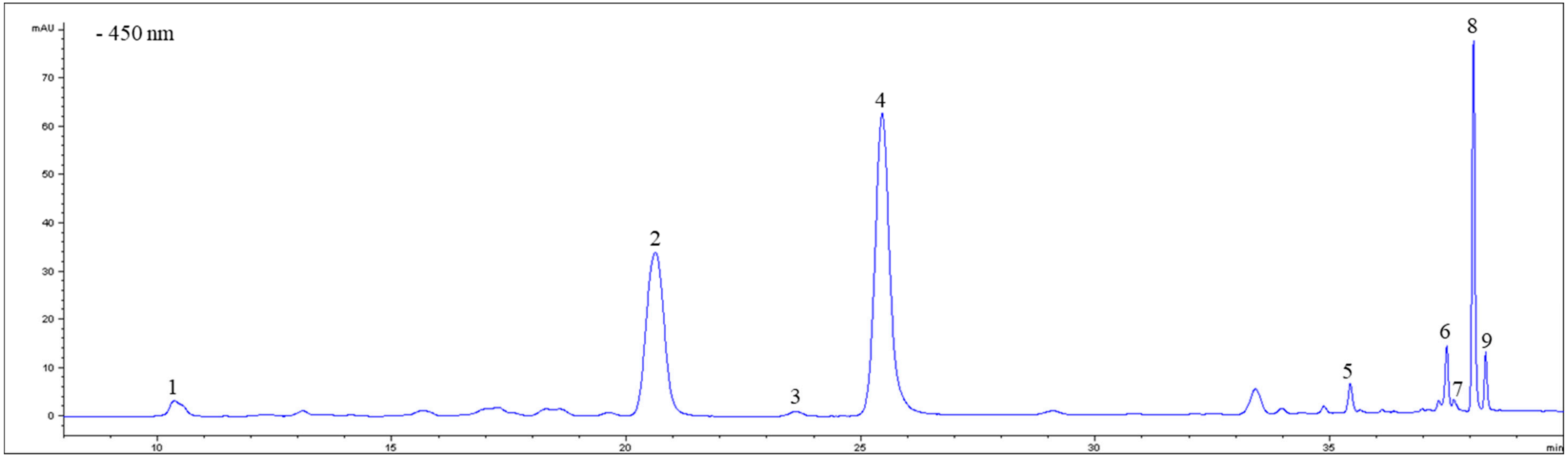

Figure S3. HPLC chromatogram of glucosinolates in radish sprouts (0 DAI) grown under nitrogen sufficient condition. 1, Progoitrin; 2, Sinigrin; 3, Glucoalyssin; 4, 4-Hydroxyglucobrassicin; 5, Glucobrassicanapin; 6, Glucoraphasatin; 7, Glucobrassicin; 8, 4-Methoxyglucobrassicin; 9, Neoglucobrassicin.

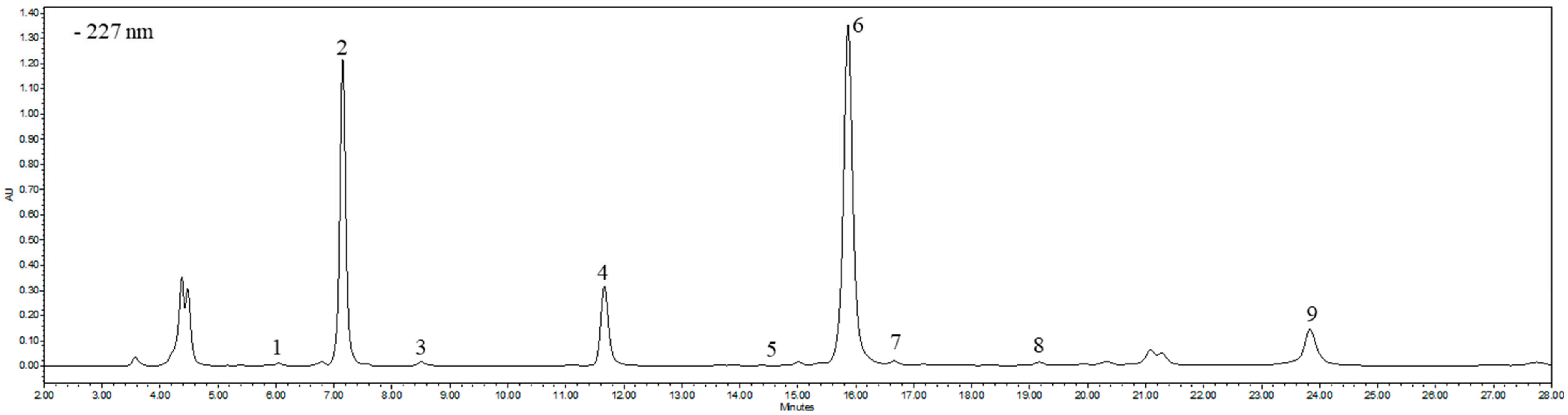

Figure S4. Metabolite expression data mapped onto the schematic pathway of radish sprouts grown under (A) nitrogen sufficient and (B) nitrogen deficient conditions. The log2 fold change (log_2 FC) values of metabolite levels relative to the levels at 0 day after light incubation began (DAI). The log_2 FC values are represented as a gradient and can be visualized per box on the top right (increased abundance is shown red and decrease in abundance is shown in green). The gray boxes represent the metabolites that could not be detected. The solid-lines represent a direct link and dotted-lines represent an indirect link between the metabolites. The solid-line arrows show the transfer of metabolites to cell organelles. G6P, Glucose 6-phosphate; F6P, Fructose 6-phosphate; F1,6BP, Fructose 1,6-bisphosphate; 3PG, 3-Phosphoglyceric acid; PEP, 2-Phosphoenolpyruvate; MEP, Mevalonate; MVA, Mevalonic acid.

(A)

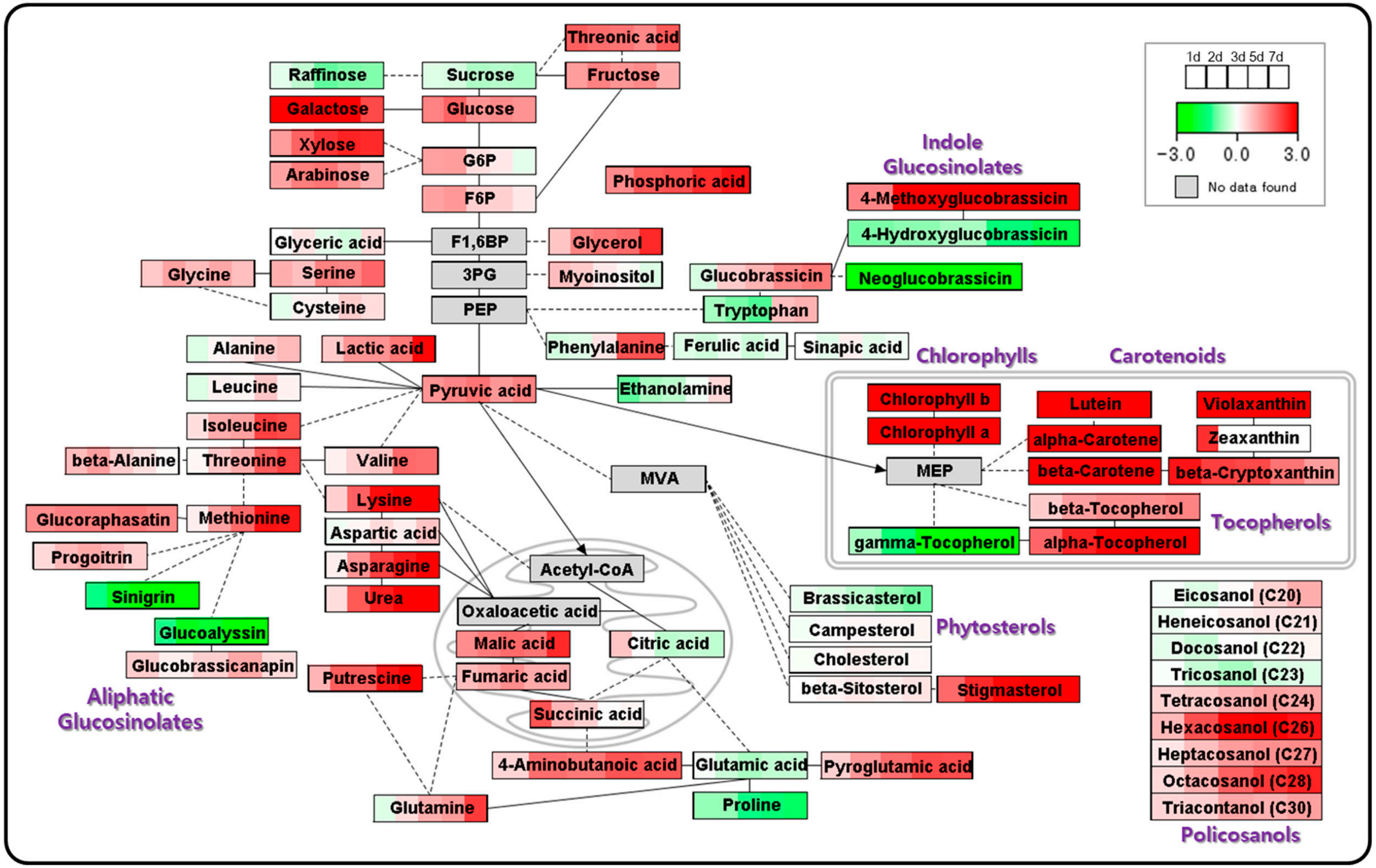

Figure S4. (*Continued*)

(B)

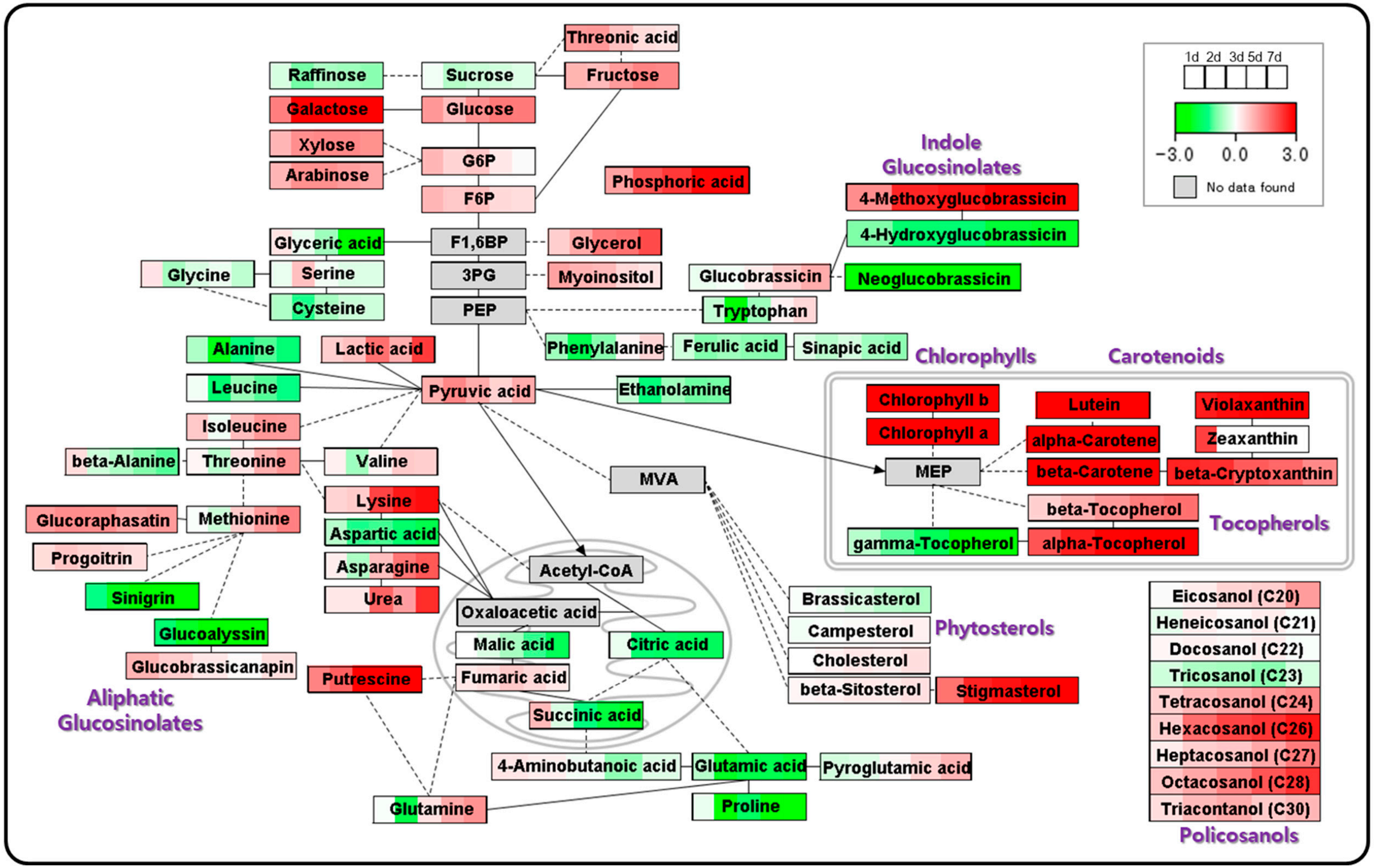

Supplement: Supplementary file 1 [file plants-08-00361-s001.pdf]
